# Supplementary material for: Netcombin: An algorithm for constructing optimal phylogenetic network from rooted triplets
Source: PLoS One. 2020 Sep 18;15(9):e0227842. doi: 10.1371/journal.pone.0227842 (PMC7500971; doi:10.1371/journal.pone.0227842)
Supplement: S4 File — (ZIP) [file pone.0227842.s004.zip › SequenceData-Triplet/sequenceData-10-20-30-0-Triplet/length 200, numseq 30, rate 10^-7/6 seq.pdf]

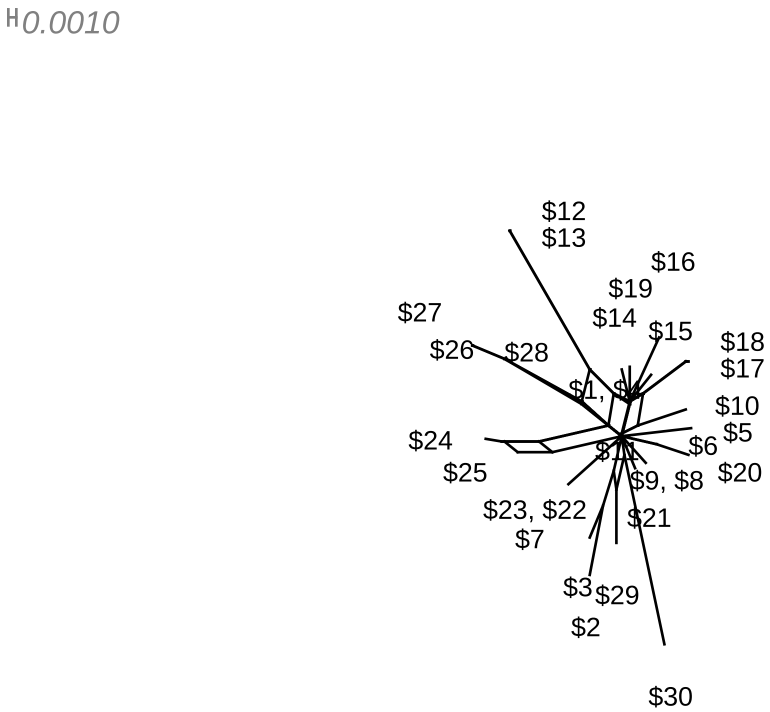
0.0010
$12
$13
$16
$19
$14
$15
$18
$17
$27
$26
$28
$1,
$10
$5
$6
$24
$11
$25
$9, $8
$20
$23, $22
$21
$7
$3
$29
$2
$30
